# Efficacy and safety of endostar combined with chemoradiotherapy versus chemoradiotherapy alone in locally advanced cervical cancer: A PRISMA-compliant systematic review and meta-analysis

**DOI:** 10.1097/MD.0000000000030170

**Published:** 2022-09-09

**Authors:** Nuersimanguli Maimaitiming, Xiaoli Ma, Yu Wei, Leiyu Cao, Yan Gao, Li Zhang

**Affiliations:** a Department of Four Comprehensive Internal Medicine, The First Affiliated Hospital of Xinjiang Medical University, Xinjiang, People’s Republic of China.

**Keywords:** antivascular targeted therapy, cervical cancer, curative effect, endostar, meta analysis

## Abstract

To evaluate the role and safety of endostar in cervical cancer by comparing the efficacy and adverse reactions of endostar combined with concurrent chemoradiotherapy in patients with locally advanced cervical carcinoma.

**Methods:**

The quality of the included literature was evaluated by searching the database for the comparison of endostar combined with concurrent radiotherapy and chemotherapy in cervical cancer patients; objective response rate (ORR) and disease control rate (DCR) were used as the main outcome indicators, and statistical analysis was performed using RevMan5.3 and State15.3 software.

**Results:**

A total of 13 studies were included in this study, including 1057 patients with locally advanced cervical cancer, suggesting that endostar combined with chemoradiotherapy can significantly improve the objective response rate (ORR: odds ratio 3.88, 95% confidence interval 2.77–5.45, *P* < .00001) and disease control rate (DCR: odds ratio 4.43, 95% confidence interval 2.78–7.04; *P* < .00001), and there was no significant increase in treatment-related adverse reactions.

**Conclusions:**

In this meta-analysis, endostar combined with concurrent chemoradiotherapy significantly improved ORR and DCR in patients with locally advanced cervical cancer without increasing toxicity. However, this study only analyzed the short-term efficacy of endostar, and its influence on overall survival and progression-free survival needs to be further verified in large randomized controlled trials with long-term follow-up.

## 1. Introduction

Cervical cancer is the fourth leading cause of cancer-related deaths among women in the world.^[1]^ In 2020, there were approximately 604,000 new cases and 342,000 deaths worldwide, among which 48.6% of new cases and 55.5% of deaths occurred in Asia, posing a serious threat to women’s health.^[2]^ In recent years, with the widespread application of cervical cytology screening and inoculation with human papillomavirus vaccine, cervical cancer and precancerous lesions can be detected and treated early, resulting in a yearly decline in the prevalence of cervical cancer.^[3]^ Unfortunately, due to limited economic and health conditions, similar improvements have not been made in developing countries, where 87% of cervical cancer-related deaths still occur.^[4]^ The pathological types of cervical cancer mainly include squamous cell carcinoma, adenocarcinoma, adenosquamous carcinoma, and other rare types, and squamous cell carcinoma accounts for about 80% of them.^[5]^ In the treatment of early cervical cancer, radical surgery plus other adjuvant therapies can significantly relieve the patient’s condition, or even achieve complete remission (CR).^[6]^ According to the statistics, the 5-year survival rate of patients with early cervical squamous cell carcinoma is 75%. For patients with locally advanced cervical cancer (stage IIB–IV), concurrent chemoradiotherapy (CCRT) plus brachytherapy is still the standard treatment in the globally and China.^[7]^ The disease control rate (DCR) of patients with locally advanced cervical cancer is significantly lower than early stage cervical cancer, and there is a higher risk of recurrence and metastasis, so the treatment of locally advanced cervical cancer still needs to be further explored.

It is found that tumor angiogenesis is closely related to tumor genesis, development, invasion, and metastasis. Be different from normal angiogenesis, tumor angiogenesis is an uncontrolled process. Since the needs of rapid tumor cell proliferation surpass the capacity of host vasculature, hypoxia and low supplies of nutrients characterize early stages of tumor development.^[8]^ Hypoxia triggers the expression of pro-angiogenic factors such as vascular endothelial growth factor (VEGF) and platelet-derived growth factor (PDGF), which promotes tumor angiogenesis.^[9]^ Therefore, targeted communication between tumor cells and adjacent vasculature is the basis of antitumor angiogenesis therapy. Multiple studies have demonstrated that the use of anti-VEGF targeted drugs in solid tumors can significantly prolong the overall survival. Thus, VEGF has become 1 of the ideal targets for tumor therapy.^[10]^ Based on this, antivascular targeting drugs such as bevacizumab came into being.

Bevacizumab, a recombinant humanized monoclonal antibody against VEGF, has been used to treat patients with advanced cervical cancer in recent years.^[11]^ It inhibits biological activity by competitively binding with VEGF, inhibited endothelial cell proliferation and neovascularization, promoted vascular normalization, increased the intratumor infiltration through VEGF/VEGFR2 pathway, so as to achieve antitumor effect. However, many studies have shown that bevacizumab has a variety of side effects and strict indications for clinical use, thus limiting its wide clinical application.^[12]^In contrast, recombinant human endostatin (endostar), as an emerging anti-VEGF targeted tumor angiogenesis drug independently developed in China, has shown good application prospects in lung cancer, melanoma, nasopharyngeal cancer, and gastric cancer.^[13]^

Compared with bevacizumab, endostar has higher stability and longer half-life period, and can inhibit tumor vascular growth through multiple targets. In addition, endostar can help normalize tumor vascular network, improve hypoxia microenvironment, and enhance the effect of antitumor therapy.^[14]^ A randomized controlled trial (RCT) study found that endostar showed good efficacy in patients with locally advanced cervical squamous cell carcinoma.^[15]^ However, the efficacy and safety of endostar combined with CCRT in patients with locally advanced cervical cancer have not been systematically evaluated. Therefore, this study aims to compare the efficacy and safety of endostar combined with CCRT versus chemoradiotherapy alone in locally advanced cervical cancer through systematic review and meta-analysis, and provide theoretical basis for the treatment of locally advanced cervical cancer patients.

## 2. Methods

### 2.1. Registration

This study has been developed in strict accordance with the priority report item for systematic review and meta-analysis (PRISMA) criteria and has been registered with PROSPERO at CRD42021275316.

### 2.2. Search strategy

This study performs the search strategy and selection process according to PRISMA criteria. PubMed, Medline, Web of Science, EMBASE, Cochrane Library, CNKI, Wanfang, VIP, and other databases were searched by entering keywords into the system. This meta-analysis includes RCT, nonrandomized controlled study (NRCT), and cohort study with a publication deadline of September 26, 2021; Chinese key words are “locally advanced,” “cervical cancer,” “endostar,” “endostar” or “recombinant human endostar,” “concurrent chemoradiotherapy” or “chemoradiotherapy”; The English keywords are “cervical cancer” or “cervical carcinoma” and “endostar,” “endostar” or “recombinant human endostatin injection” and “concurrent” Chemoradiotherapy “or” chemoradiotherapy. In addition, we reviewed other relevant studies and literatures to find more potential articles.

### 2.3. Study selection

#### 2.3.1. Eligibility criteria.

Inclusion criteria: All patients were histopathologically diagnosed as locally advanced cervical cancer; The study design was RCT, NRCT, or cohort study; The purpose of the study was to compare the efficacy and safety of endostar combined with CCRT; and All patients with cervical cancer were treated for the first time; and Study outcome indicators should include at least the following 3 types: Objective response rate (ORR); DCR; and Adverse events.

Exclusion criteria: Studies with design defects and poor quality; Insufficient survival data; Research not based on humans; Studies on patients with various organ dysfunction; and Single-arm clinical studies, case-control studies, reviews, case reports, meta-analysis, and duplicate data.

### 2.4. Data extraction

The 2 researchers conducted literature screening and data extraction in strict accordance with inclusion and exclusion criteria. When there arose any disagreement, we consulted with the third reviewer to reach a consensus. We extracted the following information from each study: general information (including first author name, publication year, study time, and region), patient pathology type, clinical stage, number of patients in experimental and control groups, concurrent chemotherapy regimen and radiotherapy dose, treatment-related side effects, and clinical outcomes.

### 2.5. Assessment of methodological quality

For the included RCTs, the quality of the included studies was assessed using the Cochrane Systematic Evaluation Manual 5.3, which included 7 aspects: randomized controlled studies, allocation concealment, blinding of participants and researchers, blinding of outcome evaluation, completeness of outcome data, selective reporting, and other biases. Based on the evaluation results, the included literature was classified as “high risk,” “low risk,” and “unknown,” and the possibility of various biases was minimized if the above criteria were fully satisfied. If the above-mentioned criteria were fully satisfied, the possibility of bias was minimal and the grade was A. If the above-mentioned criteria were partially satisfied, the possibility of bias was moderate and the grade was B. If the above-mentioned criteria were not satisfied, the possibility of bias was high and the grade was C.

The quality of the included NRCT trials and cohort studies was evaluated using the Cohort Study Newcastle–Ottawa Scale (NOS) scale, which included: (1) study population: representativeness of the exposed group (1 point), representativeness of the nonexposed group (1 point), determination of exposure factors (1 point), and determination of outcome indicators not yet to be observed at the start of the study (1 point); (2) comparability between groups: comparability between exposed and nonexposed groups was considered in the design and statistical analysis (2 points); and (3) outcome measures: adequacy of the study for outcome evaluation (1 point), adequacy of follow-up time (1 point), adequacy of follow-up in exposed and nonexposed groups (1 point), total 9 points, 1–3 for low quality, 4–6 for moderate quality, and 7–9 for high quality.

### 2.6. Statistical method

Revman 5.3 and State 15.3 software were used to analyze the collected data. For dichotomous variables such as ORR and DCR, and the incidence of adverse reactions, odds ratio (OR), and its 95% confidence interval (CI) were used as effect analysis statistics, while for continuous variables, mean difference and its 95% CI were used as effect analysis statistics. *P* < .05 was considered statistically significant, and α = 0.05 was the test level. *I*^2^ > 50% indicates significant heterogeneity. The random effect model is adopted, and the source of heterogeneity needs to be further determined by subgroup analysis; otherwise, the fixed effect model is adopted. In addition, State15.3 software was used for sensitivity analysis of included studies to determine the impact of each study on meta-analysis results, and publication bias was evaluated by funnel plot and Begg and Egger tests.

## 3. Results

### 3.1. Study selection

**Figure 1.**
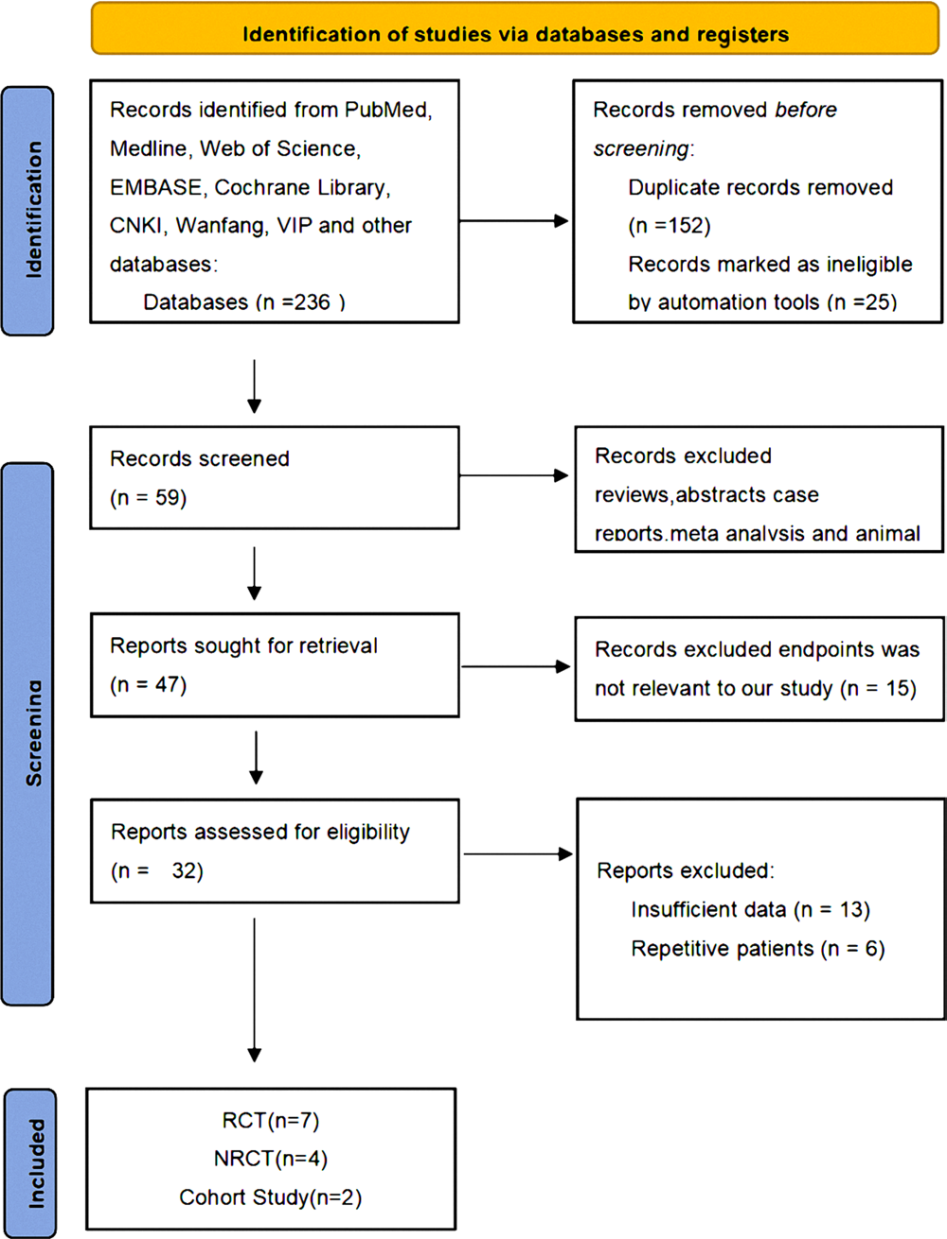
Flow diagram of literature search and selection.

According to the above retrieval strategy, a total of 236 studies were related to the efficacy and safety of endostar combined CCRT in cervical cancer. Firstly, 152 duplicated literatures were excluded. Secondly, through reading titles and abstracts, 52 articles were excluded because that did not satisfy the inclusion criteria, such as reviews, case reports, animal experiments, and meta-analysis. Then, 19 literatures with incomplete survival data and low quality were further excluded after reading full text. Finally, 13 studies (a total of 1057 patients with cervical cancer) were included in our study, including 7 RCTS, 4 NRCT, and 2 cohort studies (Fig. 1). The above studies all researched that the efficacy and safety of endostar combined with CCRT vs CCRT alone in local advanced cervical cancer. Partial response (PR), complete response (CR), stable disease (SD), progressive disease (PD), ORR, DCR, and adverse events were reported in all included studies. Progression-free survival (PFS) was reported in 1 RCT study, 1 NRCT study, and 1 cohort study. But due to different experimental methods, the influence on PFS cannot be compared. The basic characteristics of the included studies are shown in Table 1.

**Table 1:** Intervention characteristics of included studies.

Study/ID	Hospitalization time	Type	Histological type (observation/control)			Clinical stage	N (observation/control)	Regimen	
			Squamous	Adeno	Adenosquamous			Observation	Control
Lu et al^[16]^	2017.01–2020.01	RCT	66/32	12/6	0	IB2–IVA	78/38	E + CCRT (cisplatin, qw)	CCRT (cisplatin, qw)
Chen and Fu^[17]^	2016.02–2018.02	NRCT	49/46	11/10	0	IIB–IVA	60/56	E + CCRT (TP)	CCRT(TP)
Dili et al^[18]^	2015.01–2017.05	Cohort study	54/58	24/28	0	IIB–IVA	78/86	E + CCRT (DDP, qw)	CCRT (DDP, qw)
Tang^[19]^	2017.07–2018.11	NRCT	27/27	3/3	0	IIB–IVA	30/30	E + CCRT (DDP, qw)	CCRT (DDP, qw)
Xu et al^[20]^	2014.02–2016.05	RCT	26/28	6/6	0	IIB–IVA	32/34	E + CCRT (DDP, qw) + thermotherapy	CCRT (DDP, qw) + thermotherapy
Fan et al^[21]^	2013.02–2014.02	RCT	20/21	8/7	0	IIB–IVA	28/28	E + CCRT (DDP, qw)	CCRT (DDP, qw)
Luo^[22]^	2011.01–2014.01	RCT	20/22	13/11	0	IIB–IVA	33/33	E + CCRT (liposome, qw)	CCRT (liposome, qw)
Liu et al^[23]^	2007.01–2008.12	NRCT	25/25	0	0	III bulky	25/25	E + CCRT (DDP, qw) + SC (TP)	CCRT (DDP, qw) + SC (TP)
Ke et al^[24]^	2009.10–2010.1	RCT	18/19	8/7	0	IIB–IVA	26/26	E + CCRT (DDP, qw)	CCRT (DDP, qw)
Shu et al^[15]^	2019.06–2020.12	RCT	48/43	0	0	IB3–IVA	48/43	E + CCRT (TP)	CCRT (TP)
Zhao et al^[25]^	Unknown	RCT	15/15	5/5	5/5	IIB–IV	25/25	E + CCRT (DDP qw + Hu po bid)	CC (DDP qw + Hu po bid)
Zhang^[26]^	2010.2–2013.6	Cohort study	49/40	8/6	0	IIB–IVA	57/46	E + CCRT (DDP, qw)	CC (DDP, qw)
Li^[27]^	2017.1–2020.2	NRCT	28/27	8/7	0	IIB–IIIB	36/34	E + CCRT (DDP, qw)	CC (DDP, qw)

CCRT = concurrent chemoradiotherapy, DDP = cisplatin, E + CCRT = endostar + concurrent chemoradiotherapy, NRCT = nonrandomized controlled trial, RCT = randomized controlled trial, TP = triptolide.

### 3.2. Baseline characteristics

**Figure 2.**
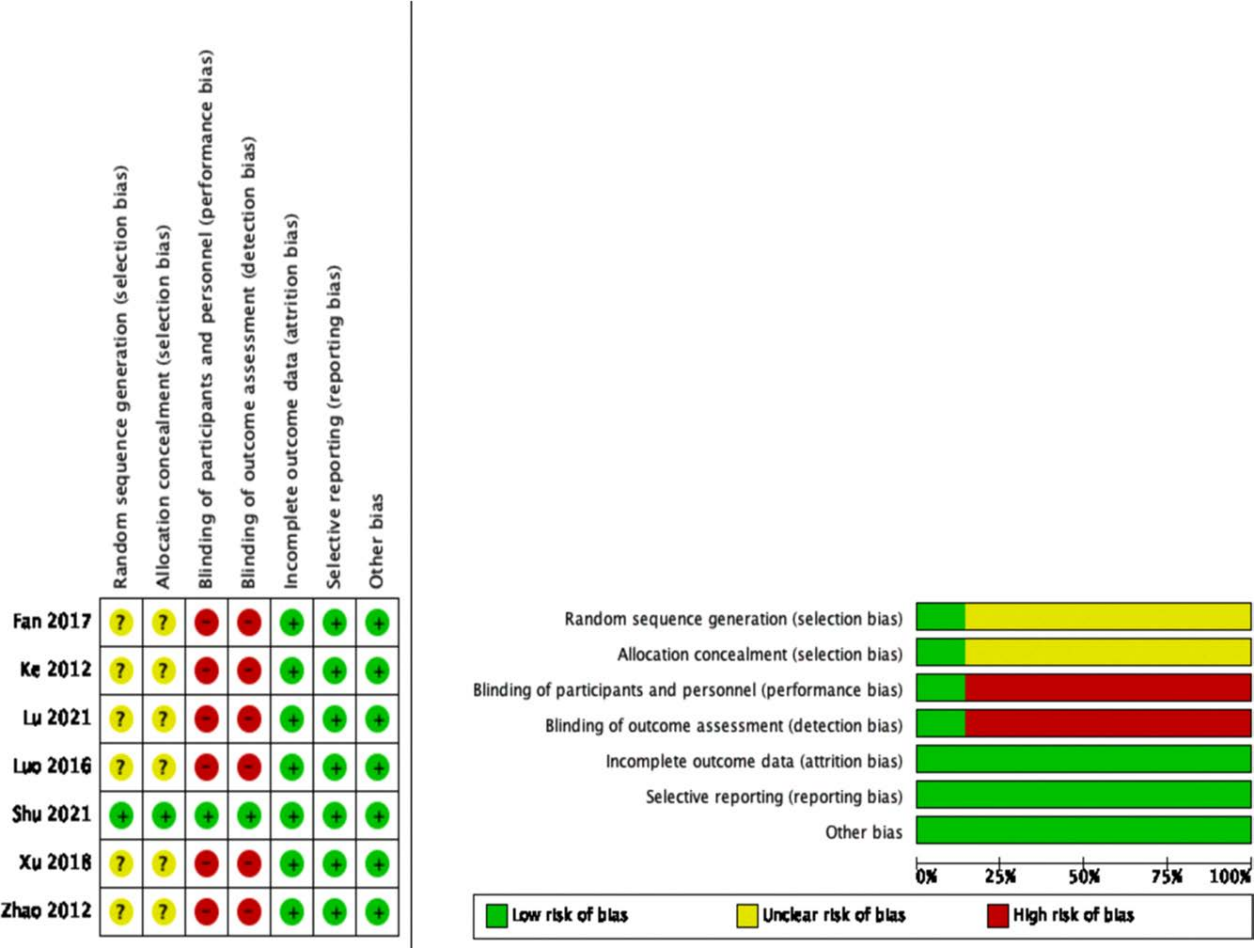
Quality assessment for RCT based on the Cochrane Systematic Review Manual 5.3. RCT = randomized controlled trial.

Among the 7 RCT studies included, 1 study used computer-generated random number classification and had a low risk of bias. The other 6 studies only described “randomized” and did not describe specific methods, so it was impossible to judge the risk of bias. One study mentioned that the random sequence was hidden and the risk of bias was low. The rest of 6 studies did not mention random sequence hiding, so the risk of bias could not be judged. One study mentioned blindness and its specific implementation methods, and the risk of bias was low. But the remaining studies did not mention blindness, and the possibility of blindness was low, so there was the high risk of bias in these studies. ORR, DCR, and adverse reactions were recorded in all included studies, and there was no statistically significant difference in baseline data, thus there was no selective reporting bias or other bias. Therefore, the results of the quality evaluation of the included RCT studies showed that, 1 was grade A and the rest were grade B (Fig. 2A). The NOS scores of 4 NRCT studies and 2 cohort studies were all >5, indicating moderate quality studies (Table 2).

**Table 2:** Risk assessment of non-RCT study bias.

	Dili et al^[18]^	Zhang^[26]^	Chen and Fu^[17]^	Tang^[19]^	Liu et al^[23]^	Li^[27]^
Representativeness of the exposed cohort	1	1	1	1	1	1
Selection of the nonexposed cohort	1	1	1	1	1	1
Ascertainment of exposure	1	1	1	1	1	1
Demonstration that outcome of interest was not present at start of study	0	0	1	1	1	1
Comparability of cohorts on the basis of the design or analysis	1	1	1	1	1	1
Assessment of outcome	0	0	0	0	0	0
Was follow-up long enough for outcomes to occur	1	1	0	0	1	0
Adequacy of follow-up of cohorts	1	1	0	0	1	0
Total	6	6	5	5	7	5

RCT = randomized controlled trial.

### 3.3. Results of meta-analysis

#### 3.3.1. Objective response rate.

**Figure 3.**
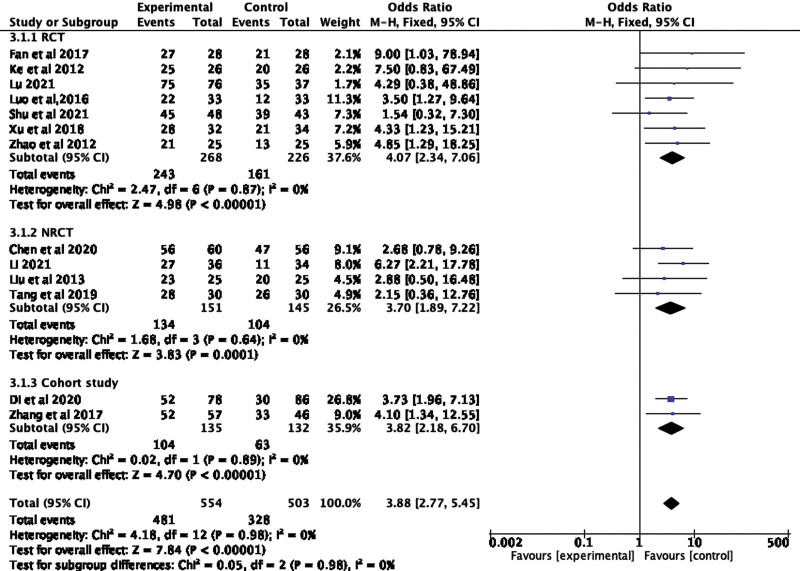
Forest plot of ORR for CCRT + endostar versus CCRT with different study designs. CCRT = concurrent chemoradiotherapy, ORR = objective response rate.

The 13 included studies recorded ORR values, and the unrecorded ones were calculated by (CR + PR)/total number of cases × 100%. The above data were meta-analyzed by Revman5.3 software. The result showed that endostar combined with CCRT significantly increased ORR (OR 3.88, 95% CI 2.77–5.45; Heterogeneity test: *I^2^* = 0%, *P* < .00001, fixed effect model), no heterogeneity between these studies. The results of our studies suggested that the ORR of the experimental group was higher than that in control group. We further conducted subgroup analysis of the included literature based on the types of trials, and the results were consistent with the previous results. In other words, endostar combined with CCRT can significantly improve patients’ ORR compared with CCRT alone no matter the test method (RCT: *P* < .00001, NRCT: *P* = .0001, Cohort study: *P* < .00001), the difference was statistically significant. The specific results are shown in Figure 3.

#### 3.3.2. Disease control rate.

**Figure 4.**
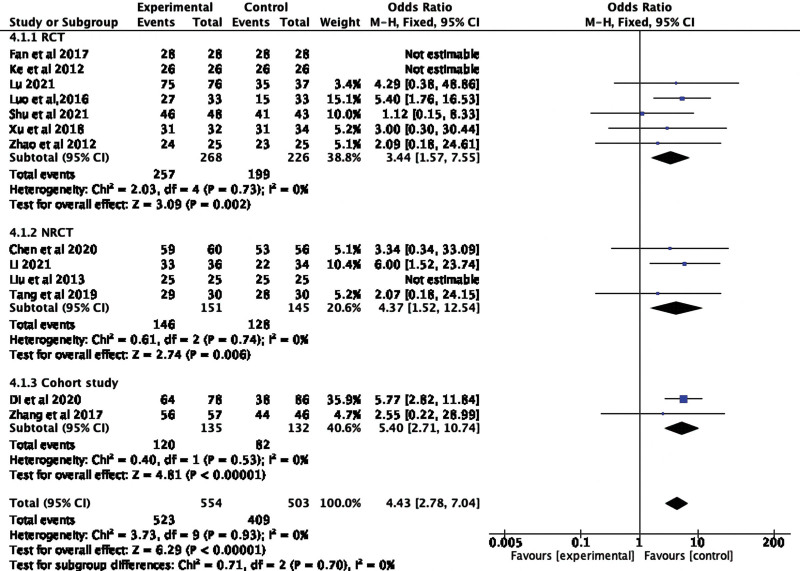
Forest plot of DCR for CCRT + endostar versus CCRT with different study designs. CCRT = concurrent chemoradiotherapy, DCR = disease control rate.

DCR were recorded in all 13 included studies, and the unrecorded ones were calculated by (CR + PR + SD)/total number of cases × 100%. The above data were meta-analyzed by Revman5.3 software, too. Meta-analysis showed that endostar combined with CCRT significantly increased the DCR (OR 4.43, 95% CI 2.78–7.04; Heterogeneity test: *I^2^* = 0%, *P* < .00001, fixed effect model), no heterogeneity between these studies. We further conducted a subgroup analysis of the included studies based on the different test method, and the results showed that endostar combined with CCTR in RCT, NRCT, and cohort studies could significantly improve patients’ DCR (RCT: *P* = .002, NRCT: *P* = .006, cohort study: *P* < .00001), the difference was statistically significant. The results are shown in Figure 4.

### 3.4. Adverse reactions

The National Cancer Institute Toxicity Criteria 4.0 were used to assess the adverse reactions of antitumor treatment. Table 3 shows the safety outcomes between endostar combined CCRT and CCRT alone. We summarized adverse reactions caused by endostar combination therapy according to the type of trial. The results showed that endostar combined CCRT could reduce the probability of neutropenia in RCT (OR = 0.36, 95% CI: 0.15–0.85, *P* < .05), but can increase incidence rate of hypertension (OR = 10.80, 95% CI: 1.25–93.44, *P* < .05). In the NRCT group, endostar combined CCRT did not increase the treatment-related adverse reactions of patients compared with the CCRT group, and the differences were not statistically significant. In cohort studies, endostar combined CCRT increased the probability of diarrhea and radiation proctitis （*P* < .05), but only 1 study reported these adverse effects. However, all the above-mentioned adverse reactions can be completely improved after active intervention. Among them, hypertension is the most recognized adverse reaction of endostar, which needs to pay attention in the treatment process. At present, there were no serious adverse reactions caused by endostar combined CCRT in patients with locally advanced cervical cancer.

**Table 3:** Treatment-related adverse reactions.

Side effect	RCT	NRCT	Cohort study
	Estimate (95% CI)	Estimate (95% CI)	Estimate (95% CI)
Myelosuppression	—	OR 1.26 (0.75–2.09)	OR 1.15 (0.19–6.94)
Leukopenia	OR 0.78 (0.24–2.49)	—	—
Neutropenia	OR 0.36(0.15–0.85)^*^	—	—
Thrombocytopenia	OR 1.08 (0.55–2.14)	OR 0.71 (0.20–2.51)	—
Anemia	OR 0.72 (0.28–1.81)	OR 0.75 (0.23–2.44)	—
Bleeding	OR 1.68 (0.63–4.49)	—	—
cardiovascular toxicity	OR 6.60 (0.77–56.65)	—	—
Liver and kidney dysfunction	OR 0.79 (0.21–3.03)	OR 1.10 (0.58–2.09)	—
Neurotoxicity	—	—	OR 1.75 (0.56–5.48)
Gastrointestinal reaction	OR 0.80 (0.37–1.71)	OR 1.24 (0.74–2.10)	OR 2.15 (0.42–11.03)
Nausea	OR 0.74 (0.32–1.68)	—	—
Vomiting	OR 0.85 (0.34–2.10)	—	—
Diarrhea	OR 0.81 (0.45–1.46)	—	OR 4.53 (1.60–12.84)^**^
Radiation proctitis	OR 1.67 (0.49–5.72)	OR 0.95 (0.47–1.91)	OR 0.25 (0.10–0.66)^**^
Radiation cystitis	OR 2.29 (0.20–26.22)	OR 1.00 (0.18–5.51)	—
Rash	OR 1.49 (0.55–4.04)	—	—
Fever	OR 1.67 (0.42–6.61)	—	—
Alopecia	OR 1.46 (0.43–4.93)	OR 0.78 (0.25–2.39)	OR 2.89 (0.91–9.17)
Hypertension	OR 10.80 (1.25–93.44)^*^	—	—
Genitourinary tract reaction	OR 1.22 (0.14–11.01)	OR 0.87 (0.30–2.48)	OR 1.50 (0.27–8.28)

CI = confidence interval, NRCT = nonrandomized controlled trial, OR = odds ratio, RCT = randomized controlled trial.

*P < 0.05, **P < 0.01.

### 3.5. Sensitivity analysis and publication bias

**Figure 5.**
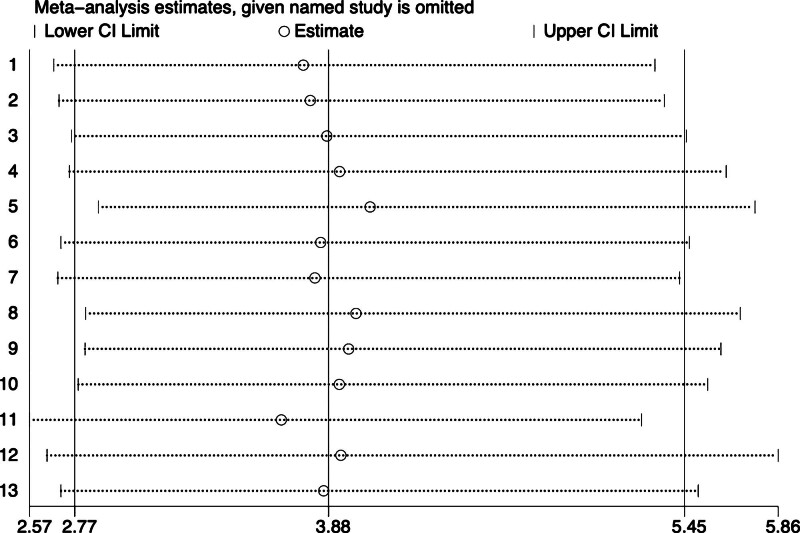
Sensitivity analyses.

**Figure 6.**
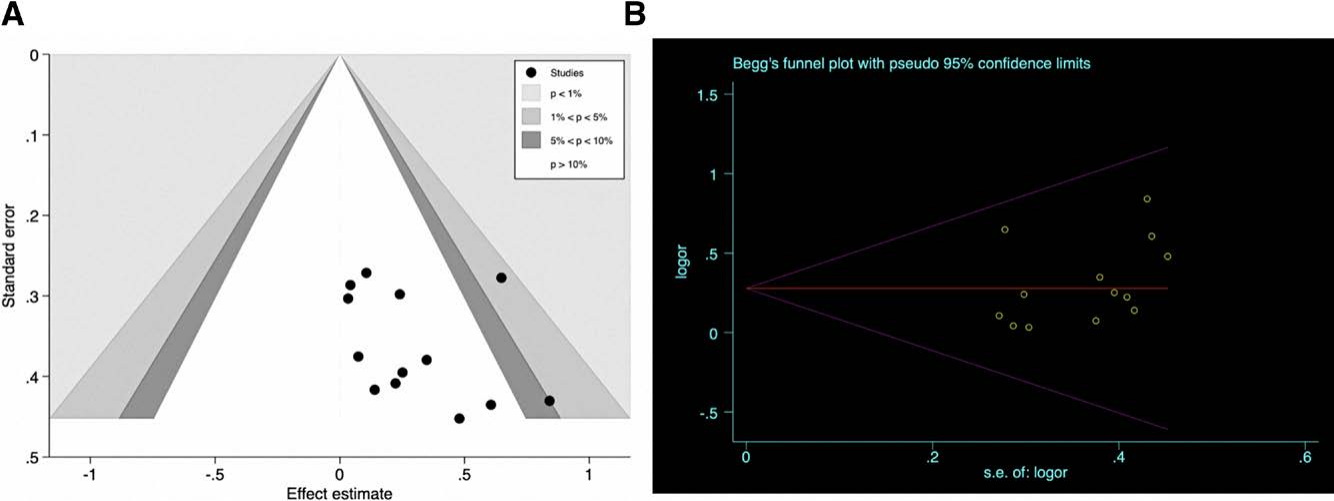
The results of publication bias. (A) Enhanced funnel plot. (B) Begg funnel plot.

Sensitivity analysis found there was no significant change in size or direction after analyzed studies excluded one by one, suggesting that there’s no significant heterogeneity among these studies, and the stability of the study was acceptable (Fig. 5). In view of publication bias, the enhanced funnel plot in State 15.3 software was used to qualitatively analyzed the included studies, and the results of enhanced funnel plot showed that the distribution of studies was relatively symmetric (Fig. 6A). To clarify publication bias in further step, Begg test (*P* = .855) and Egger test (*P* = .280) were used to further quantitatively analyze the included studies. The *P* values were all above .05, suggesting that there was no significant publication bias among these studies (Fig. 6B).

## 4. Discussion

CCRT is an internationally accepted treatment for locally advanced cervical cancer. Because of the malignant tumor cell proliferation, the tumor will grow rapidly in a short period of time, and the growth of blood supply vessels is relatively lagging, which further leads to ischemia and hypoxia of tumor tissues and cells, thus reducing the lower radiation sensitivity.^[28]^ The study of Gray et al^[29]^ showed that there was a certain correlation between partial pressure of oxygen and radiation sensitivity in tumor tissue, that is, radiotherapy sensitivity increased with partial pressure of oxygen, and when the oxygen partial pressure goes up higher, the tumor tissues will be more sensitive to radiotherapy. Many studies have also shown that oxygen partial pressure mainly depends on the perfusion function of blood vessels, and a key marker of malignant tumors is continuous abnormal angiogenesis. Abnormal neovascularization not only promotes the occurrence and development of invasive tumors, but also constructs abnormal tumor microenvironment.^[30]^ Secondly, hypoxia can induce the expression of multidrug resistence-1 (MDR-1), increase the resistance of tumor cells to chemotherapy drugs, change the cycle of tumor cells, and make most tumor cells stop in G1 phase.^[31]^ Hypoxia can also reduce the expression of topoisomerase II in tumor cells, resulting in drug resistance of chemotherapeutic drugs metabolized by topoisomerase. As mentioned above, abnormal structure and function of neovascularization network in tumor result in reduced the chance of tumor cells contacting with chemotherapy drugs.^[32]^ At the same time, hypoxia leads to apoptosis inhibition of tumor cells, and apoptosis induction is one of the important mechanisms of chemotherapeutic drugs, so hypoxia can increase chemotherapeutic resistance.^[33]^ Viallard and Larrivée^[34]^ and others researchers described the relationship between the abnormal tumor angiogenesis and tumor microenvironment. The morphology and structure of tumor neovascularization become highly disordered under the continuous action of angiogenic factors such as VEGF. This results in increased interstitial hydraulic pressure, hypoxia, decreased pH, and abnormal tumor microenvironment with immunosuppression, resulting in tumor resistance to radiotherapy and chemotherapy and reduced the antitumor treatment efficacy.

VEGF is the key mechanism leading to highly abnormal tumor microenvironment. Therefore, anti-angiogenic therapy blocking VEGF may break this vicious cycle, reshape abnormal tumor microenvironment, improve cell hypoxia, and increase the sensitivity and efficacy of radiotherapy and chemotherapy drugs.^[35]^ Endostar is an angiogenesis inhibiting neovascularization antitumor targeted drug developed by Chinese scientists in 1999. Its mechanism of action is to inhibit abnormal angiogenesis by targeting VEGF, improve the abnormal microenvironment inside tumor tissues, and reduce the proliferation and metastasis of tumor tissues.^[36]^ It can improve the sensitivity of radiotherapy and chemotherapy and further improve the prognosis of patients. In a nude mouse model inoculated with Hela cells in the abdomen, endostar inhibited the proliferation and metastasis of the transplanted tumor by inhibiting angiogenesis and lymphatic vessels.^[37]^ Meanwhile, endostar combined with CCRT showed the best inhibitory effect on tumor growth. It may be related to endo-induced apoptosis of tumor cells, down-regulated expression of VEGF and hypoxic inducible factor (HIF-1α).^[38]^ Recently, Li et al^[39]^ conducted a phase II clinical trial in patients with locally advanced cervical cancer, when VEGFR2 expression was positive, the short-term ORR and DMFS in patients with endostar combined with CCRT were significantly longer than those in patients with CCRT alone (*P* < .05), and there was no significant increase in treatment-related adverse reactions (*P* > .05). Another study^[15]^ showed that endostar combined with CCRT improved the disease remission rate of patients in a certain extent. However, the incidence of adverse reactions such as cardiac adverse reaction, digestive system reaction and allergy is correspondingly increased, so the indication of the use of endostar is not clear. In addition, there is no systematic review to evaluate the efficacy of endostar in locally advanced cervical cancer, and its clinical application is still controversial. Therefore, in this study, we systematically collected relevant clinical studies and conducted a meta-analysis to explore the efficacy and safety of endostar combined CCRT in patients with locally advanced cervical cancer.

A total of 13 studies (including RCT, NRCT, and cohort studies) were included in this study, and the results showed that endostar combined with CCRT in the treatment of locally advanced cervical cancer could significantly improve the ORR and DCR of patients (*P* < .05), with no significant heterogeneity. These results suggest that the combination of endostar can improve the short-term efficacy of local advanced cervical cancer patients. The results of sensitivity analysis showed that the stability of this study was fair and the publication bias was small. This study comprehensively included relevant studies, providing a strong evidence for the clinical application of endostar in patients with locally advanced cervical cancer.

In addition, the combined use of endostar may increase the occurrence of treatment-related adverse reactions in patients to a certain extent. Analysis of adverse reactions in this study showed that endostar combined with CCRT significantly increased the occurrence of gastrointestinal adverse reactions in patients compared with the control group. Studies showed that endostar combined treatment could increase the incidence of hypertension in patients, and there was no statistically significant difference in the incidence of other adverse reactions between the experimental group and the control group, and no treatment-related deaths were reported. Previous studies have reported that endostar in advanced lung squamous cell carcinoma are more prone to cardiotoxicity-related adverse reactions, mainly manifested as increased heart rate, palpitations, abnormal T waves and ST-T changes.^[40]^ In contrast, there was no relevant reports reported in patients with locally advanced cervical cancer. However, the changes of cardiac enzymes, electrocardiogram, and cardiac ultrasound should be paid attention to in the process of application of endostar, and the cardiac function of patients should be closely observed. Other mentioned adverse reactions, such as hematotoxicity, abnormal liver and kidney function, neurotoxicity, radiation-related adverse reactions, urogenital reactions, and other adverse reactions, showed no statistical difference, but could not be ignored in clinical application.

This study reported the efficacy and safety of endostar combined with CCRT versus CCRT alone in locally advanced cervical cancer through meta-analysis, aiming to provide theoretical basis for clinical treatment of cervical cancer. The shortcomings of this study are as follows: There are few researches included in this study, most of which are single-center and small-sample studies; The types of studies included were RCT, NRCT, and cohort studies. Among the 7 included RCT studies, only 1 study reported the specific implementation methods of random allocation, allocation hiding, and blindness. The included NRCTs and retrospective cohort studies were of medium quality, and the overall quality of enrolled studies was not high; and This study only reported the short-term efficacy of endostar in locally advanced cervical cancer, and its long-term efficacy in patients with cervical cancer needs to be further proved. Endostar, as a novel antivascular treatment option, still has many problems to be solved. Big sample size and high-quality studies are needed to confirm the better efficacy and safety of endostar combination therapy.

## 5. Conclusion

Degree of what has been discussed above, endostar not only in the microscopic molecular level on endothelial cell surface protein expression and regulation of intracellular signaling pathways include macro on the reshaping of the tumor microenvironment, which in turn improve the curative effect of comprehensive treatment of tumor. The ORR and DCR of patients can be significantly improved in the treatment of locally advanced cervical cancer, providing theoretical basis for its clinical application. At present, although there is low quality evidence supporting the prolongation of overall survival and PFS in patients with endostar combined CCRT, this result has not been confirmed, and needs to be further confirmed by large RCTs with long-term follow-up.

## Author contributions

NM collected and analyzed the data, wrote the paper. XM and YW analyzed the data. LC and YG conceived and designed this study. LZ designed the structure of this article and reviewed the final manuscript prior to submission. All authors read and approved the final manuscript.

**Conceptualization:** Yu Wei.

**Data curation:** Nuersimanguli Maimaitiming.

**Formal analysis:** Xiaoli Ma.

**Investigation:** Leiyu Cao.

**Investigation:** Xiaoli Ma.

**Methodology:** Yu Wei.

**Resources:** Yan Gao.

**Writing – original draft:** Nuersimanguli Maimaitiming.

**Writing – review & editing:** Li Zhang.

## References

[1] Tornesello ML Faraonio R Buonaguro L. The role of microRNAs, long non-coding RNAs, and circular RNAs in cervical cancer. Front Oncol. 2020;10:150. 32154165 10.3389/fonc.2020.00150 PMC7044410

[2] Sung H Ferlay J Siegel RL. Global cancer statistics 2020: GLOBOCAN estimates of incidence and mortality worldwide for 36 cancers in 185 countries. CA Cancer J Clin. 2021;71:209–49. 33538338 10.3322/caac.21660

[3] El-Zein M Richardson L Franco EL. Cervical cancer screening of HPV vaccinated populations: cytology, molecular testing, both or none. J Clin Virol. 2016;76(suppl 1):S62–8. 26631958 10.1016/j.jcv.2015.11.020 PMC4789074

[4] Guo M Xu J Du J. Trends in cervical cancer mortality in China from 1989 to 2018: an age-period-cohort study and Joinpoint analysis. BMC Public Health. 2021;21:1329. 34229639 10.1186/s12889-021-11401-8 PMC8259057

[5] Nishio M To Y Maehama T. Endogenous YAP1 activation drives immediate onset of cervical carcinoma in situ in mice. Cancer Sci. 2020;111:3576–87. 32716083 10.1111/cas.14581 PMC7541006

[6] Kilic F Cakir C Yuksel D. Analysis of the prognostic factors determining the oncological outcomes in patients with high-risk early-stage cervical cancer. J Obstet Gynaecol. 2022;42:281–8. 33938363 10.1080/01443615.2021.1882974

[7] Todo Y Watari H. Concurrent chemoradiotherapy for cervical cancer: background including evidence-based data, pitfalls of the data, limitation of treatment in certain groups. Chin J Cancer Res. 2016;28:221–7. 27199520 10.21147/j.issn.1000-9604.2016.02.10 PMC4865615

[8] Eckert F Zwirner K Boeke S. Rationale for combining radiotherapy and immune checkpoint inhibition for patients with hypoxic tumors. Front Immunol. 2019;10:407. 30930892 10.3389/fimmu.2019.00407 PMC6423917

[9] Lee P Chandel NS Simon MC. Cellular adaptation to hypoxia through hypoxia inducible factors and beyond. Nat Rev Mol Cell Biol. 2020;21:268–83. 32144406 10.1038/s41580-020-0227-y PMC7222024

[10] Cervantes-Villagrana RD Albores-García D Cervantes-Villagrana AR. Tumor-induced neurogenesis and immune evasion as targets of innovative anti-cancer therapies. Signal Transduct Target Ther. 2020;5:99. 32555170 10.1038/s41392-020-0205-z PMC7303203

[11] Klein A Loewenstein A. Therapeutic monoclonal antibodies and fragments: bevacizumab. Dev Ophthalmol. 2016;55:232–45. 26502311 10.1159/000431199

[12] Shu Q Li W Li H. Vasostatin inhibits VEGF-induced endothelial cell proliferation, tube formation and induces cell apoptosis under oxygen deprivation. Int J Mol Sci. 2014;15:6019–30. 24722573 10.3390/ijms15046019 PMC4013612

[13] Xu F Ma Q Sha H. Optimizing drug delivery for enhancing therapeutic efficacy of recombinant human endostatin in cancer treatment. Crit Rev Ther Drug Carrier Syst. 2007;24:445–92. 18197781 10.1615/critrevtherdrugcarriersyst.v24.i5.20

[14] Liu X Nie W Xie Q. Endostatin reverses immunosuppression of the tumor microenvironment in lung carcinoma. Oncol Lett. 2018;15:1874–80. 29434884 10.3892/ol.2017.7455 PMC5774419

[15] Shu H Dong Y Xu Z. The efficacy and safety of continuous intravenous endostar treatment combined with concurrent chemoradiotherapy in patients with locally advanced cervical squamous cell carcinoma: a randomized controlled trial. Front Oncol. 2021;11:723193. 34485157 10.3389/fonc.2021.723193 PMC8414882

[16] Lu H Wu Y Liu X. Endostar, an antiangiogenesis inhibitor, combined with chemoradiotherapy for locally advanced cervical cancer. Oncol Res. 2022;28:929–44. 34544526 10.3727/096504021X16318716607908 PMC8790112

[17] Chen J Fu H. The efficacy of endostar combined with radiotherapy and chemotherapy and its effects on imbalance of CD4+ T cells of advanced cervical cancer patients. Zhongliu Yaoxue Zazhi. 2020;10:709–13.

[18] Dili S Aisi T Zhao H. Analysis of the efficacy and safety of concurrent chemoradiotherapy combined with recombinant human endostatin in the treatment of advanced cervical cancer. Zhongguo Yixue Qianyan Zazhi. 2020;12:61–5.

[19] Tang Y. The study of endostar on sensitization of radiotherapy for cervical cancer. Dalian Medical University. 2019.

[20] Xu X Wen S Wang H. Clinical study of endostar combined with hyperthermiaand radiochemotherapy for the treatment of advanced cervicalcancers. Xiandai Yangsheng. 2018:39–40.

[21] Fan Y Yu Z Cui X. Recombinant human endostar combined with chemoradiotherapy Therapeutic effect analysis of cervical cancer. Zhongguo Yaowu Yu Linchuang. 2017;17:865–68.

[22] Luo J. Effect of recombinant human endostatin in adjuvant treatment of advanced cervical cancer. Shandong Yiyao. 2016;56:72–4.

[23] Liu X Hou B Xiaojun S. Endostar degrees with chemoradiation therapy period giant block type cervical cancer clinical curative effect observation. Wujing Houqin Xueyuan Xuebao. 2013;22:178–81.

[24] Ke Q Zhou S Du W. Chemoradiation joint human recombinant endostatin treatment for locally advanced cervical cancer efficacy analysis of 52 cases. Shiyong Aizheng Zazhi. 2012;27:373–5.

[25] Zhao J Dong X Zhao J. Human recombinant endostatin combined hydroxyurea chemoradiation curative effect observation of treatment of locally advanced cervical cancer. Zhongguo Shiyong Yiyao. 2012;7:65–6.

[26] Zhang Y. Concurrent chemoradiotherapy combined therapy with recombinant human endostatin middle-late analysis of the clinical effect and prognosis of cervical cancer. Shanxi Yiyao Zazhi. 2017;46:1711–4.

[27] Li J. The effect of ondo combined with concurrent radiotherapy and chemotherapy in the treatment of locally advanced cervical cancer and its effect on serum VEGF,SCCA, CYFRA21-1. Shiyong Aizheng Zazhi. 2021;36:1136–9.

[28] Chaudary N Hill RP Stulik L. The oral CXCR4 inhibitor X4-136 improves tumor control and reduces toxicity in cervical cancer treated with radiation therapy and concurrent chemotherapy. Int J Radiat Oncol Biol Phys. 2021;110:1317–24. 33771702 10.1016/j.ijrobp.2021.03.031

[29] Gray SL Hall KE Powell LL. Tissue oxygen saturation in dogs with acute hemorrhage. J Vet Emerg Crit Care (San Antonio). 2018;28:408–14. 30117666 10.1111/vec.12752

[30] Abou Khouzam R Brodaczewska K Filipiak A. Tumor hypoxia regulates immune escape/invasion: influence on angiogenesis and potential impact of hypoxic biomarkers on cancer therapies. Front Immunol. 2021;11:613114. 33552076 10.3389/fimmu.2020.613114 PMC7854546

[31] Shen X Zhi Q Wang Y. Hypoxia induces multidrug resistance via enhancement of epidermal growth factor-like domain 7 expression in non-small lung cancer cells. Chemotherapy. 2017;62:172–80. 28351036 10.1159/000456066

[32] Korbecki J Kojder K Barczak K. Hypoxia alters the expression of CC chemokines and CC chemokine receptors in a tumor-A literature review. Int J Mol Sci . 2020;21:5647. 32781743 10.3390/ijms21165647 PMC7460668

[33] Yart L Bastida-Ruiz D Allard M. Linking unfolded protein response to ovarian cancer cell fusion. BMC Cancer. 2022;22:622. 35672715 10.1186/s12885-022-09648-4 PMC9172076

[34] Viallard C Larrivée B. Tumor angiogenesis and vascular normalization: alternative therapeutic targets. Angiogenesis. 2017;20:409–26. 28660302 10.1007/s10456-017-9562-9

[35] Hang X Zhao L Wu B. BCL-2 isoform β promotes angiogenesis by TRiC-mediated upregulation of VEGF-A in lymphoma. Oncogene. 2022;41:3655–63. 35701534 10.1038/s41388-022-02372-0

[36] Zhang G Jin G Nie X. Enhanced antitumor efficacy of an oncolytic herpes simplex virus expressing an endostatin-angiostatin fusion gene in human glioblastoma stem cell xenografts. PLoS One. 2014;9:e95872. 24755877 10.1371/journal.pone.0095872 PMC3995956

[37] Jia Y Liu M Cao L. Recombinant human endostatin, Endostar, enhances the effects of chemo-radiotherapy in a mouse cervical cancer xenograft model. Eur J Gynaecol Oncol. 2011;32:316–24. 21797125

[38] Lin K Ye P Liu J. Endostar inhibits hypoxia-induced cell proliferation and migration via the hypoxia-inducible factor-1α/vascular endothelial growth factor pathway in vitro. Mol Med Rep. 2015;11:3780–5. 25543905 10.3892/mmr.2014.3131

[39] Li Y Tian Y Jin F. A phase II multicenter randomized controlled trial to compare standard chemoradiation with or without recombinant human endostatin injection (Endostar) therapy for the treatment of locally advanced nasopharyngeal carcinoma: long-term outcomes update. Curr Probl Cancer. 2020;44:100492. 32035692 10.1016/j.currproblcancer.2019.06.007

[40] Zhang X. Endostar combined GP scheme used for patients with advanced non-small cell lung cancer clinical treatment effect. Zhongguo Baojian Yingyang. 2020;30:78.

